# Prevalence of late-stage presentation and associated factors of cervical cancer patients in Tikur Anbesa Specialized Hospital, Ethiopia: institutional based cross-sectional study

**DOI:** 10.1186/s13027-021-00371-6

**Published:** 2021-05-11

**Authors:** Mulugeta Wassie, Beletech Fentie

**Affiliations:** 1 School of Nursing, College of Medicine and Health Sciences, University of Gondar, Gondar, Ethiopia; 2 Department of Pediatrics and Child Health Nursing, School of Nursing, College of Medicine and Health Sciences, University of Gondar, Gondar, Ethiopia

**Keywords:** Cervical cancer, Late-stage presentation, Ethiopia

## Abstract

**Background:**

Cancer of the uterine cervix remains a main public health problem in Sub-Saharan Africa. About two-thirds of patients with cervical cancer were diagnosed at late stage with contributing factors of out-of-pocket medical bill, looking for care out of conventional health settings and multiple visits to healthcare facilities before diagnostic confirmations in Addis Ababa. Therefore, the aim of this study was to identify prevalence of late-stage presentation and associated factors among cervical cancer patients in Tikur Anbesa Specialized Hospital (TASH).

**Methods:**

Institutional based cross-sectional study was conducted from March to April 2019 in TASH oncology center. Data were extracted from patient’s chart using structured checklist prepared in English and analyzed using STATA14.2. Binary logistic regression model was used to identify variables that affect the outcome variable.

**Results:**

A total of 1057 cervical cancer patients were included in this five-years retrospective study. The prevalence of late-stage presentation among cervical cancer patients was 56.8%. It was affected by being anemic [AOR = 1.55,95%CI (1.17–2.10)], came from Oromia region (AOR = 0.65,95%CI (0.46–0.91) and Addis Ababa city [AOR:0.5;95%CI (0.34–0.73)], rural residency [AOR:1.88;95% (1.38–2.56)] and age ≥ 60 years [AOR:1.89;95%CI (1.12–3.20)].

**Conclusion:**

The study revealed that the prevalence of late-stage presentation among cervical cancer patients is high. Being anemic, regions where patients came from, rural residency and age group ≥ 60 years were statically significant. It is better to expand cervical cancer education for rural dwellers, expand cancer treatment centers and prioritize to patients with anemia and advanced age.

## Background

Cervical cancer remains a major public health problem in Sub-Saharan Africa. Predictions of the future years are also very frightening. Many challenges are associated with cervical cancer care in low- and middle-income countries including insufficient number of trained health care personnel, limited facilities and geopolitical factors such as wars, environmental disasters and HIV pandemic. Recent advances in molecular biology techniques make it possible to carry out a very early diagnosis of human papilloma virus (HPV) infections of genital organs among the women up to 30 years old [1, 2].

Two very effective prevention strategies for cervical cancer are vaccination against HPV and cervical cancer early screening with primary HPV testing followed by treatment of detected precancerous lesions. Realizing quick scale-up of immunization and two times lifetime uterine cervix screening in the world could prevent up to 13.4 million uterine cervix malignancy over the future half century [2, 3].

On the study conducted in Addia Ababa, the median health-seeking and diagnostic intervals of women with cervical malignancy were ten and ninety-seven days respectively with 75% of delayed diagnostic confirmation. Nearly 60% of patients with cervical cancer were diagnosed at late stage with the significant factors of out-of-pocket medical bill, looking for care out of conventional health care areas and several visits to health settings before diagnostic confirmations [4–6].

The etiology of cervical cancer was thought due to breaching social taboos or undertaking unacceptable behaviors and the supposed benefits of modern treatment were very low. Traditional remedies were the most preferred treatment options for early stage of the disease though most cases presented at advanced stage in which treatment options are ineffective. Limited awareness and inaccessibility of appropriate health services were independent predicting factors of late-stage presentation [7].

Nearly 64% cervical cancer patients were diagnosed with late-stage of the disease (IIIB-IVB) [8]. About 45%of cervical cancer patients presented with late stage (FIGO Stage III) and most (91.4%) histology type was squamous cell carcinoma. The five-year survival across stages was 50% with predictor of FIGO staging (62% stage II vs 45% stage III) [9–13]. Therefore, the aim of this study was to identify prevalence of late-stage presentation and associated factors among cervical cancer patients in Ethiopia.

## Methods

### Study design, period and area

Institutional based cross-sectional study was conducted in the oncology center of TASH, Ethiopia, from March to April 2019. TASH is the biggest referral public hospital in Ethiopia established in 1972. It is the training center of health professionals with undergraduate and postgraduate programs and others paramedics. The hospital is staffed by many health professionals from various disciplines. It has over 800 beds and the beds reserved for cancer care are 20. It is the only oncology center in Ethiopia and the only cancer registry center for Addis Ababa.

### Populations

All medical records of the women diagnosed with cervical cancer in TASH cancer center from January 1, 2014 to December 31,2018 were study populations. Cervical cancer patients with medical charts that were incomplete and not found during data collection were excluded in the study.

### Sample size and study variables

All women with cervical malignancy diagnosed at TASH beginning January 2014 to December 2018 was the sample size. Profiles of all women with cervical malignancy diagnosed and treated from January 2014 to December 2018 were evaluated and 1057 medical records that met the inclusion criteria were selected. Stage at presentation of cervical cancer was the outcome variable whereas sociodemographic, pathological and clinical characteristics were explanatory variables.

### Operational definitions

Stage at diagnosis: the revised FIGO staging for carcinoma of the vulva, cervix, and endometrium was used [14].

Late stage: late stage at presentation was to mean patients presented with FIGO stages III and IV.

Anemia: patients’ hemoglobin level below 12.0 g/dl was classified as anemic [15].

Comorbidity: The presence of any conditions (mentioned in the Carlson comorbidity Index [16] other than cervical cancer at diagnosis.

Substance use: Patients who used one, two or all of the three substances (cigarate, chat and alcohol) [17].

### Data collection tools and quality assurance

Data was collected from the patients’ medical records using structured check list. The checklist consisted three parts: sociodemographic, pathological and clinical characteristics. Two master’s holders in clinical oncology nursing supervised the data collection process and three nurses with bachelors of science in nursing were data extractors. Pretest of the checklists was done to test the checklists’ reliability with real data collection and essential modifications were made accordingly.

### Data processing and analysis

The extracted data were entered and checked using Epi-data 3.1, then it was exported in to Stata14.2 for analysis. Frequencies, proportions and descriptive statistics were used to explain the study population with the relevant variables. Binary logistic regression was used to analyze factors that affect the outcome variable. Variables with *p*- value < 0.2 with bivariable analysis were included to multivariable analysis. Explanatory variables which have *p*-value < 0.05 in multivariable analysis were identified as a significant factor affecting the outcome variable (late stage) with 95% confidence level.

### Ethical clearance

Ethical approval for this study was obtained from the Institutional Review Boards of school of nursing and midwifery, Addis Ababa University. The letter of permission was written from school of nursing and midwifery to the oncology center of TASH. Then, the oncology center chief administrator permitted to collect the data. The study was conducted without individual informed consent since data extraction were relied on chart review other than patients.

## Result

### Sociodemographic characteristics of cervical cancer patients

This study was conducted among 1057 cervical cancer patients presented within five consecutive years in oncology center of TASH. The mean age of study participants was 50 years with a minimum and maximum ages of 15 and 87 years respectively. More than half (58.1%) of the patients came from urban area and about 26.5% of the patients came from Addis Ababa city. Nearly one sixth (14.4%) were governmental workers, about two third (62.9%) were married and 17.2% were substance users (Table 1).

**Table 1:** Socio demographic characteristics of cervical cancer patients in TASH oncology center, Ethiopia (*n* = 1057)

Variables	Code	Early stage		Late stage	
		Frequency	(%)	Frequency	(%)
Age	< 30	45	48.9	47	51.1
	30–39	100	45.7	119	54.3
	40–49	103	39.3	159	60.7
	50–59	111	45.3	134	54.7
	≥ 60	98	41.0	141	59.0
Residency	Urban	315	51.3	299	48.7
	Rural	142	32.1	301	68.0
Region	Amhara	97	34.5	184	65.5
	Oromia	149	43.7	192	56.3
	Tigray	8	26.7	22	73.3
	SNNP	31	32.6	64	67.4
	Addis Ababa	161	57.5	119	42.5
	Others	11	36.7	19	63.3
Religion	Orthodox	267	42.4	363	57.8
	Muslim	87	43.5	113	56.5
	protestant	99	46.7	113	53.3
	Others	6	40.0	9	60.0
Occupation	Employed	73	48.0	79	52.0
	Unemployed	384	42.4	521	57.6
Marital status	Married	287	43.2	378	56.8
	unmarried	170	43.4	222	56.6
Children	None	20	50.0	20	50.0
	One	23	33.3	46	66.7
	Two	48	32.7	99	67.4
	Three	177	49.0	184	51.0
	> three	189	43.0	251	57.0
Substance	User	81	44.5	101	55.5
	None user	376	43.0	499	57.0

### Clinical characteristics of cervical cancer patients

**Fig. 1:**
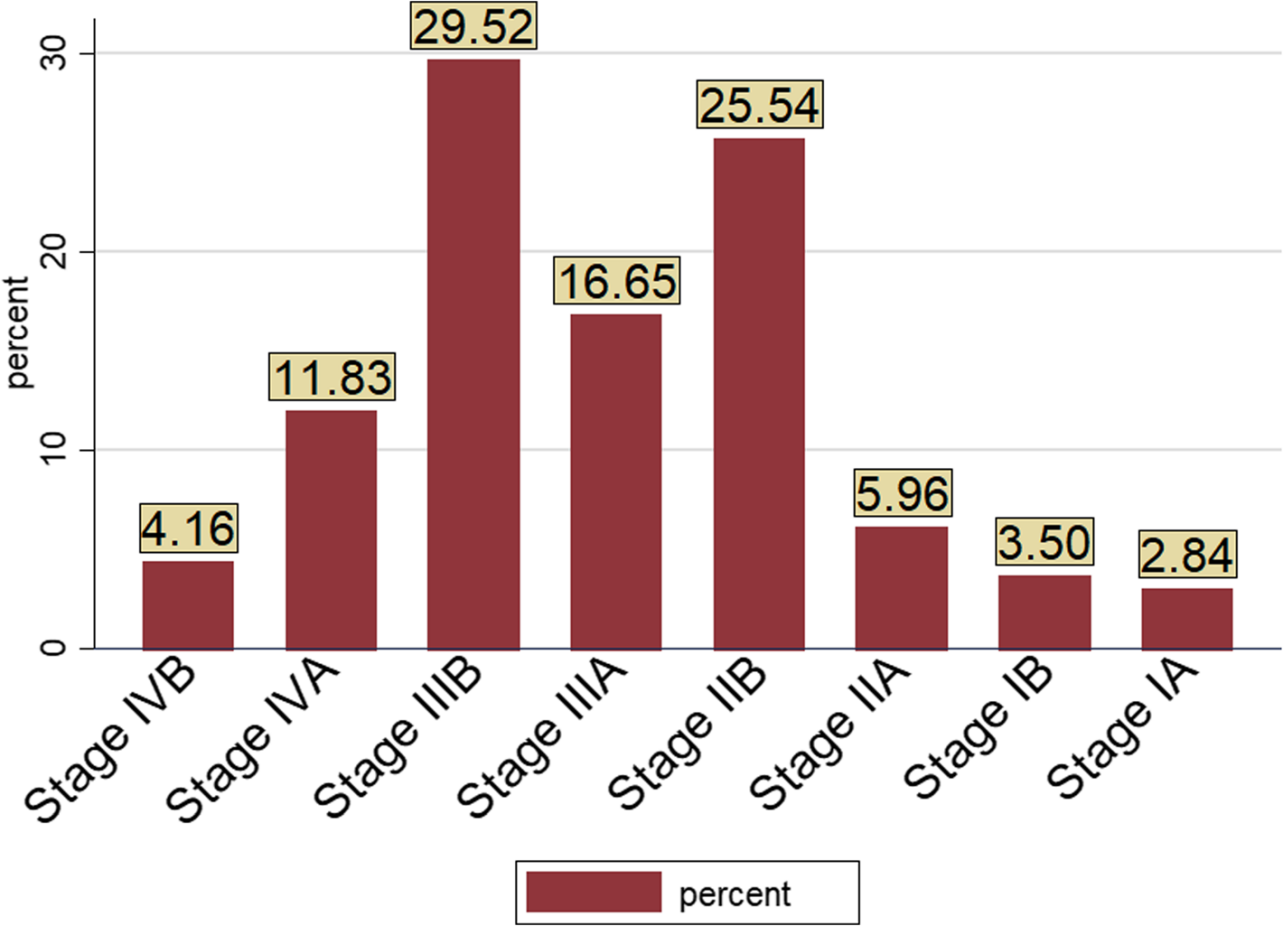
FIGO Stages at presentation of cervical cancer patients in TASH oncology center, Ethiopia (*n* = 1057)

Nearly one third (32.5%) of patients had comorbidity at presentation and about 18.4% were HIV positive. Histologically, most (91%) cervical cancer patients had squamous cell carcinoma (Table 2). Based on FIGO staging, about 3% of patients were with stage IA and nearly 30% patients were with stage IIIB (Fig. 1).

**Table 2:** Clinical characteristics of study participants in TASH oncology center, Ethiopia (*n* = 1057)

Variables	Code	Early stage		Late stage	
		Frequency	(%)	Frequency	(%)
Comorbidity	Yes	142	41.3	202	58.7
	No	315	44.2	398	55.8
Anemia	Yes	210	38.8	331	61.2
	No	247	47.9	269	52.1
Histology type	Squamous cell	414	43.2	545	56.8
	Adenocarcinoma	43	43.9	55	56.1
HIV status	Negative	383	44.4	480	55.6
	Positive	74	38.1	120	61.9

### Prevalence of late-stage presentation among cervical cancer patients

The prevalence of Late-stage presentation among cervical cancer patients was 56.8% [95%CI (53.6–59.7)]. About 60.7% of patients with late stages were within 40–49 age categories and about 68% came from rural area. Nearly three quarter (73.3%) of late-stage patients came from Tigray region and about 42.5% were from Addis Ababa city. Around 58% of late-stage patients were none governmental employees and 56.8% were married. Roughly 56% of substance users presented with late stage, 59% of patients with comorbidity came at late stage and nearly 62% of HIV positive patients presented with late stage. Nearly 56.8% of patients with squamous cell carcinoma and 61%of anemic patients presented with late stage (Tables 1 and 2).

### Factors of late-stage presentation among cervical cancer patients

Independent variables were analyzed individually with the outcome variable and the variables with the *p*- < 0.2 were included in the multivariable binary logistic regression.

In multivariable binary logistic regression analysis, anemia, region, residency and age of the patients significantly affected the outcome variable (late-stage presentation) at *p*-value < 0.05 with 95% CI.

The study revealed that cervical cancer patients with anemia presented with late stage 1.55 times [AOR = 1.55,95%CI (1.17–2.1)] more than who weren’t anemic. Patients who came from Oromia region were at 35% [AOR = 0.65,95% CI (0.46–0.913)] lower presented with late stage than patients who came from Amhara regional state. Similarly, patients who came from Addis Ababa city were at 50% [AOR:0.5;95%CI (0.337–0.729)] lower than those who came from Amhara regional state to present with late stage. Patients living in rural area presented with late stage 1.88 times [AOR:1.88;95% (1.381–2.56)] more than urban dwellers. Study Patients with age groups ≥ 60 years presented with late stage 1.89 times [AOR:1.89;95%CI (1.115–3.199] more than those with age group < 30 years as a reference (Table 3).

**Table 3:** Result of multivariable analysis of factors contributing to late-stage presentation of cervical cancer patients in TASH oncology center, Ethiopia (*n* = 1057)

Variables	Codes	AOR	*P*-value	95% CI
Comorbidity	No		1	
	Yes	0.95	0.798	0.65–1.39
Anemic status	No		1	
	Yes	1.55	**0.002**	1.17–2.05
Marital status	Unmarried		1	
	Married	0.97	0.834	0.74–1.28
No. of children	None		1	
	One	1.63	0.251	0.71–3.74
	Two	1.65	0.187	0.78–3.48
	Three	0.76	0.432	0.38–1.52
	more than 3	0.97	0.934	0.48–1.95
Substance	None user		1	
	User	0.92	0.673	0.64–1.33
Occupation	None worker		1	
	Gov’t worker	0.91	0.626	0.62–1.33
Region	Amhara		1	
	Oromia	0.65	**0.013**	0.46-.91
	Tigray	1.25	0.620	0.52–3.02
	SNNP	1.02	0.940	0.61–1.71
	Addis Ababa	0.500	** < 0.001**	0.34-.73
	Others	0.93	0.865	0.41–2.13
Resident	Urban		1	
	Rural	1.88	** < 0.001**	1.38–2.56
Age	< 30		1	
	30–39	1.35	0.261	0.79–2.29
	40–49	1.50	0.124	0.89–2.51
	50–59	1.35	0.258	0.80–2.26
	≥ 60	1.89	**0.018**	1.12–3.19
HIV status	Positive		1	
	Negative	0.78	0.288	0.49–1.24

N.B:1 = reference category

## Discussion

This study focused on the late-stage presentation and associated factors of cervical cancer patients in Ethiopia. In this study, about 57% cervical cancer patients presented with late stage. The current prevalence was lower than the studies conducted in Addis Ababa [4], Ghana [18], Tanzania [8], Nigeria [19] and Malaysia [20] with the prevalence’s of 60.4, 65.97, 63.9, 71.8 and 60% respectively. But it is higher than the studies conducted in Morocco [21], India [9], England [22] and Mexico [23] with their prevalence’s of 39.9, 45.4, 28 and 17.8% respectively. The possible justification of the differences in prevalence could be due to sample size, study period and the accesses of cervical cancer information differences related to Addis Ababa and health care delivery policy, socioeconomic status variations of study participants, study period, sample size and study design differences in the case of other countries mentioned other than Addis Ababa.

Anemia, region, residency and age were the major contributing factors of late-stage presentation of cervical cancer patients in the current study. Cervical cancer patients who have anemia in this study presented with late stage than those who haven’t anemia. This finding is supported by the study conducted in Tanzania [8]. This will be as being anemic could mask the signs and symptoms of the patients and leads to delay of early diagnosis. Another justification might be diagnosis of anemia in the health facilities which aren’t serving as the cancer center will delay appropriate referral as these facilities could try to treat anemia before referral. This delay of referral could associate to late-stage presentation of patients. But anemia by itself couldn’t cause late-stage presentation despite it needs further investigation.

Patients who came from Oromia region and Addis Ababa city presented relatively with early stage than patients who came from Amhara region. This disparity will be as TASH is the only oncology center in Ethiopia and very far from Amhara region and relatively nearest to Addis Ababa and Oromia regions. As a result, patients in far area could get different problems to seek oncological treatment like transportation costs and treatment costs. This is supported by different literatures conducted in different countries [24, 25].

Residency was another contributing factor of late-stage presentation of cervical cancer patient. Patients who live in the rural area presented with more late stage than those who live in the urban area in this study. This result is in agreement with different studies [26, 27]. This might be due to patients who live from urban area can get different information about the symptoms and signs of cervical cancer as compared to rural dwellers.

Cervical cancer patients with advanced age (≥ 60 years) presented with more late stage than those patients with age < 30 years. This is supported by other researchers conducted in different settings [28, 29]. This might be due to older women tend to believe that they are less vulnerable to cervical cancer and would not screen early. Another reason may be also due to older women are not seeking obstetrics and gynecology services in the post-menopausal years particularly women in rural areas where health care services are not readily accessible [30].

## Limitations

Even though this study involved relatively large sample size, establishment of a causal relationship could not be possible since it is cross sectional study. In addition, the study was conducted in one setting which couldn’t represent the whole areas of the country. Finally, the information was not recorded for the purpose of research that caused many chars with incomplete information.

## Conclusion

The study revealed that the prevalence of late-stage cervical cancer presentation is high. late-stage presentation was significantly affected by anemic status of the patients, region where the patients came from, rural residency and advanced age. It is better to give special attention to patients with anemia, advanced age, rural dwellers and those who come from remote areas. Besides, cervical cancer education could be expanded to the rural dwellers, better to increase cancer treatment centers in all regions of the country.

## Data Availability

Data will be available upon request from the corresponding author.

## Abbreviations

AOR: Adjusted Odds Ratio
FIGO: International Federation of Gynecology and Obstetrics
ASH: Tikur Anbesa Specialized Hospital
HIV: Human immune virus
HPV: Human papilloma virus
